# Early Risk Factors of Overweight Developmental Trajectories during Middle Childhood

**DOI:** 10.1371/journal.pone.0131231

**Published:** 2015-06-29

**Authors:** Laura E. Pryor, Mara Brendgen, Richard E. Tremblay, Jean-Baptiste Pingault, Xuecheng Liu, Lise Dubois, Evelyne Touchette, Bruno Falissard, Michel Boivin, Sylvana M. Côté

**Affiliations:** 1 Department of Social and Preventive Medicine, University of Montreal, Montreal, Canada; 2 Research Unit on Children’s Psychosocial Maladjustment, University of Montreal, Montreal, and University Laval, Quebec City, Canada; 3 Ste Justine Hospital Research Center, Montreal, Canada; 4 Department of Psychology, University of Quebec in Montreal, Montreal, Canada; 5 School of Public Health, Physiotherapy and Population Sciences, University College Dublin, Dublin, Ireland; 6 Institute of Genetic, Neurobiological, and Social Foundations of Child Development, Tomsk State University, Tomsk, Russian Federation; 7 Department of Epidemiology and Community Medicine, University of Ottawa, Ottawa, Canada; 8 Department of Psychoeducation, University of Quebec in Trois Rivières, Trois Rivières, Quebec; 9 National Institute of Health and Medical Research (INSERM) U669, Paris, France; 10 School of Psychology, Laval University, Quebec City, Canada; 11 International Laboratory for Child and Adolescent Mental Health Development, University of Montreal, Montreal, Canada, and French National Institute of Health and Medical Research (INSERM), Paris, France; TaipeiCityHospital, TAIWAN

## Abstract

**Background:**

Research is needed to identify early life risk factors associated with different developmental paths leading to overweight by adolescence.

**Objectives:**

To model heterogeneity in overweight development during middle childhood and identify factors associated with differing overweight trajectories.

**Methods:**

Data was drawn from the Quebec Longitudinal Study of Child Development (QLSCD; 1998-2010). Trained research assistants measured height and weight according to a standardized protocol and conducted yearly home interviews with the child’s caregiver (mother in 98% of cases). Information on several putative early life risk factors for the development of overweight were obtained, including factors related to the child’s perinatal, early behavioral family and social environment. Group-based trajectories of the probability of overweight (6-12 years) were identified with a semiparametric method (n=1678). Logistic regression analyses were used to identify early risk factors (5 months- 5 years) associated with each trajectory.

**Results:**

Three trajectories of overweight were identified: “early-onset overweight” (11.0 %), “late-onset overweight” (16.6%) and “never overweight” (72.5%). Multinomial analyses indicated that children in the early and late-onset group, compared to the never overweight group, had 3 common types of risk factors: parental overweight, preschool overweight history, and large size for gestational age. Maternal overprotection (OR= 1.12, CI: 1.01-1.25), short nighttime sleep duration (OR=1.66, CI: 1.07-2.57), and immigrant status (OR=2.01, CI: 1.05-3.84) were factors specific to the early-onset group. Finally, family food insufficiency (OR=1.81, CI: 1.00-3.28) was weakly associated with membership in the late-onset trajectory group.

**Conclusions:**

The development of overweight in childhood follows two different trajectories, which have common and distinct risk factors that could be the target of early preventive interventions.

## Introduction

Child overweight and obesity (hereafter termed “childhood overweight”) negatively impacts physical and emotional well-being and augments the risk for adult disease [1]. It is a problem that has increased dramatically in prevalence over the past three decades [2, 3] and continues to challenge public health professionals as to how best to target its prevention [3–5]. As the likelihood overweight will persist into adulthood increases once adolescence is reached [6, 7], early prevention beginning before and during primary school is considered an important strategy [8, 9].

Increasingly, a “one size fits all” approach to prevention is acknowledged as inadequate [5], in part because of the substantial heterogeneity in childhood weight status development that has been highlighted in recent studies using a longitudinal mixture model approach [10–22]. Li and colleagues [11] identified three distinct trajectory groups characterizing the development of weight status from 2–12 years in a US sample: 1) early-onset (10.9% of children), 2) late-onset (after 8 years; 5.2%), and 3) never overweight (83.9%). This pattern has been replicated among similar age groups [12] while others have identified a fourth, decreasing group [10, 21]. In fact, up to 7 distinct trajectories have been identified [22] However, comparisons between studies are limited by differences in age range and in method for classifying overweight.

An increased understanding of the patterning and risk factors associated with distinct weight status trajectories may help to elucidate whether there exist critical periods for the risk of future overweight [18]. Furthermore, such knowledge may aid in developing preventive interventions tailored according to which risk factors are more pertinent to specific developmental paths. However, research is lacking on factors associated with differing trajectories of weight status [11, 14, 23] and studies examining heterogeneity in weight status development thus far have not controlled for a wide range of putative early life risk factors longitudinally. Doing so may aid in our understanding of the relative importance of early perinatal and childhood behavioral and environmental factors, as compared to known childhood risk factors such as parental overweight.

We examined 3 types of early life risk factors: those related to the perinatal period, those specific to the child, and those belonging to the family and social environment. The fetal and infancy periods are widely accepted as critical for exposure to occurrences promoting future overweight [24–30]. For example, there is evidence that a child’s low, and more so, high birth weight, as proxy measures for the in utero experience, may be related to later overweight [31–34]. Early childhood behavioral characteristics may also play an important role, including diet and physical activity patterns, but also television viewing [32, 35], short sleep duration [36] and emotional and behavioral problems [37, 38]. The importance of the home and social environment of the young child has also been demonstrated, including exposure to the adverse situations of family poverty [39, 40], harsh parenting [41], and peer victimization [42].

This work had two aims: 1) To estimate the heterogeneity of weight status development in the population by modeling the developmental trajectories of the probability of being overweight and; 2) To identify from among a wide set of putative perinatal, child, and family/social environment risk factors for child overweight, those which may be most likely to increase the risk that a child will follow a path leading to overweight by early adolescence.

## Methods

Data was drawn from the Québec Longitudinal Study of Child Development (QLSCD) from 1998 to 2010 [43]. A random sample of 2940 families was recruited, representing the Quebec population of families with a 5-month-old singleton infant in 1998 residing in most geographic areas of Quebec. This sample was reduced to 2120 due to non-response, inability to contact, or not meeting study criteria. A trained Research Assistant (RA) visited the participating families yearly from when the child was 5 months to 8 years old, then at 10 and 12 years. At each data collection, informed written consent was provided by all respondents. On behalf of the child, consent was given by a parent or legal guardian. The study was approved by the Health Research Ethics Committees of the Québec Statistics Institute and the University of Montreal.

Children were included in the trajectory analyses if height and weight had been measured at least once between 6 and 12 years. A weighting factor, calculated by QLSCD statisticians for use with the time 6 (age 5) data collection, was used in all analyses to approximate the initial QLSCD target population in terms of sociodemographic characteristics [44]. Of the 1678 children with at least one valid BMI data point, 48.9% had data at all 5 time points, 23.4% had data for four time points, 12% had data for three time points, 7.3% for two time points and 8.2% for one time point only. When data were weighted this sample was reduced to n = 1552.

Demographic characteristics of the weighted sample are listed in Table 1. The sample weights corrected for most socio-demographic characteristics for which there was differential attrition. However, the analysis sample still included a lower proportion of immigrants (12.8% vs 18%) and male children (52.1% vs. 64.9%) than the initial sample.

**Table 1:** Demographic Characteristics of Sample (n = 1552).

	%
Sex of child (female)	52.1
Mother born outside Canada	12.2
Mother Obtained High School Diploma	80.4
Family Income (at age 5)	
<$30,000	18.1
$30,000–60,000	36.9
>60,000	44.9
	Mean (SD)
Mother's Age at Time 1 (years)	29.2 (5.4)

### Measures

#### Outcome: Developmental trajectories of child weight status

At 6, 7, 8, 10 and 12 years, the RA weighed and measured the study child, with lightweight clothing and no shoes [45, 46]. BMI was calculated as the child’s weight in kilograms divided by height in meters squared (BMI = kg/m^2^). We created a dichotomous variable in order to classify children as overweight (= 1) or not (= 0) at each time point. The cut-offs we used to classify children as overweight were those proposed by Cole et al. [47], are specific to child sex and age in months and have been recommended for international use and comparison [47].

We chose a binary measure, rather than raw BMI or BMI z-scores, because it provides a measure of the risk for overweight that is easy to interpret by clinicians and parents. We could not use the BMI z-score measure, because the group-based developmental trajectory approach requires sufficient and interpretable variability in scores in order for trajectories to be identified. When BMI z-scores are calculated using a reference population, variance is greatly reduced such that, within each year, the BMI z-scores mean is always near zero and the standard deviation is always near 1. As a consequence, when BMI z-scores are used longitudinally, they only provide a test of change in an individuals’ relative position within a population [48] and do not allow for the identification of distinct groups with various developmental patterns in a parsimonious model. Thus, z-scores are not informative when used to model trajectories with a method designed to identify the natural variation in the distributions of scores over time.

#### Risk Factors during early childhood (0–5 years)

We examined the role of several risk factors preceding the weight status trajectories. These were conceptually relevant to the study of overweight, or found to be associated with overweight in previous studies. To test the relative importance of risk factors in different spheres of functioning and different developmental periods, we grouped factors according to 1) the prenatal and infancy period; 2) early childhood characteristics; and 3) the child’s family and social environment. RA’s collected this information during yearly interviews with the mother when the child was 5 months to 5 years old. Note that for each measurement scale used, it was standardized to a 10-point scale, and the means across all time points measured were calculated for an overall score. Unless specified otherwise, higher values indicate more problematic behaviors or situations.

#### Perinatal and infancy period (gestation and the first 1.5 years)

Birth weight (g)[49] and gestational age (weeks) were obtained from hospital records. *Size for gestational age* was calculated from these two values according to current Canadian reference values [50]. *Birth rank* was categorized as first, second, or ≥ third [11]. *Breastfeeding* (exclusive or not) was assessed when the child was 1.5 years (did not breastfeed, breastfed for < 3 months, breastfed for ≥ 3 months) [51, 52]. *Average infancy weight gain* between birth and the 1.5-year measurement was calculated as: (weight at the exact age (in months) at the 1.5 year assessment—weight at birth)/ (exact age (in months) at the 1.5 year assessment) [53, 54]. Mothers reported having *smoked during pregnancy* or suffered from *gestational diabetes*. *Parental overweight* at 1.5 years indicated whether one, both, or neither parent was classified as overweight (BMI ≥ 25 kg/m) according to self-reported height and weight [55].

#### Early childhood characteristics (1.5–5 years)

Personal characteristics examined during early childhood include the child’s early BMI (from 2.5–5 years) as well as some behavioral and emotional characteristics. The outcome variable, BMI between 6 and 12 years, was derived from objective measurements. We controlled for *Preschool BMI* (2.5, 3.5, 4.5, 5 yr) which was calculated from maternally reported height and weight values [47]. We used a dichotomous variable reflecting 1) children who did not have a history of overweight throughout preschool vs. 2) those who had a history of overweight throughout preschool. This was obtained after applying a group-based trajectory analysis to the data [56] (procedure described in *Analyses* section, paragraph 1).

Mothers rated *depressive and anxious symptoms (DAS)* and *physical aggression symptoms* (1.5, 2.5, 3.5, 4.5 and 5 yr) according to items from the Preschool Behavior Questionnaire [57, 58]. Infant *Temperament* (5 mo, 1.5 yrs) was measured using 7 items from the Infant Characteristics Questionnaire [59].

Mean total *nightly sleep duration* (2.5, 3.5, 4.5, and 5yrs) was calculated and children who slept an average of ≥10 hours were contrasted from those who slept less [36, 60]. Weekday and week-end *television viewing time* (2.5, 4.5 and 5 yr) indicated the average viewing time over the course of early childhood. *Sports* and *other lessons* (e.g. dance) at 5 years were reported as: ‘at least once per week’, ‘1 or 2 sessions’, and ‘once per month or almost never’ [45, 61]. Frequency of *free-time physical activities* was categorized as ‘at least once per week’ vs. ‘less than once per week’.

#### Child Family and Social Environment (up to 5 years)

At the 5-month assessment, mothers provided information as to their age and past mental health symptoms. *Young age of motherhood* indicated whether the mother was < 21 years at the birth of her first child [58]. *Lifetime depression* designated mothers who had ever experienced a major depressive episode [62]. Past *antisocial behaviors* indicated whether mothers engaged in ≥ 2 conduct problem behaviors before the end of high school [58]. Parenting practices were measured using 4 subscales of the *Parental Cognitions and Conduct Toward the Infant Scale* [63] at 5 months, 1.5 and 2.5 years: *affection* towards the child (5 month only), *self-efficacy*, perceived *impact*, *overprotection* and *hostile-reactive* parenting. *Maternal education* indicated whether the mother obtained a high school diploma. *Poverty* was measured as having insufficient income, using Statistics Canada’s low-income cutoffs, which consider family income in the past year, number of individuals in the household, and family location of residence (5 months, 1.5, 2.5, 3.5, 4.5, 5 yr) [64, 65]. Developmental trajectories of poverty during early childhood were modeled [66] (procedure described in *Analyses*, paragraph 1) in order to provide an overall picture of the severity of a child’s poverty history: 1) never poor (71.1%), 2) transient poverty history (15.7%) and 3) chronic poverty history (13.2%). *Family food insufficiency* (1.5, 4.5 yr) distinguished families who had experienced food insufficiency from those who had not [55]. Mothers were asked (1) ‘‘Since the birth of your child (or in the past 12 months) have you or a member of your family not eaten adequately because the family had run out of food or money to buy food?” Responses ranged from “regularly or ever month” to “no”. *Intact Family* described a situation in which the target child lived with both initial parents (5months, 1.5, 2.5, 3.5, 4.5, 5 yr), vs. having lived in a single parent or recomposed family at least once [43]. *Family functioning* (5 mo, 1.5 years) was assessed with the 12-item General Functioning Subscale of the McMaster Family Assessment Device [67, 68]. Most frequent type of *childcare* (1.5, 2.5, 3.5, 4yrs.) was categorized as either: 1) home-based; 2) cared for by relative; 3) daycare center or; 4) other [69, 70]. The following questions were asked regarding *peer victimization* at 3.5, 4.5, 5, and 6 years: In the past 6 months, how often would you say your child was (1) made fun of by other children, (2) hit or pushed by other children, (3) called names by other children? [42]. *Neighborhood safety and cohesion* (5 mo, 2.5, 4.5 yr) was derived from a five-question scale pertaining to the level of social support and mutual aid [43]. The *social problems* (5 mo, 2, 4.5 yr) measure was derived from 4 questions from a scale of neighborhood social problems, including drug and alcohol sale or use in public, and groups causing trouble [43]. *Immigrant status* was obtained by asking the mother, at 5 months, whether she or her child was or ever was considered an immigrant [71].

### Analyses

Each child was classified as overweight (= 1) or not (= 0) at each age (6–12 years) according to international cut-offs [47]. We modeled group-based developmental trajectories of weight status (overweight vs. not) from 6 to 12 years, using the semi-parametric group-based approach in the SAS (version 9.3) Proc Traj program [56]. In the case of a binary outcome, this technique yields the probability of being overweight at each age, in each group.

The model was determined without a priori hypotheses regarding the existence of distinct trajectories, their number, or shape [56]. Fit indexes (Bayesian Information Criterions (BIC), and entropy) for a two, three and four group model were compared and a joint optimization of model quality and parsimony was sought [56]. In general, the BIC increases as the number of trajectories does, whereas entropy decreases, such that the best model is usually identified by a balance between BIC and entropy. Selection was therefore guided by the importance of obtaining groups that were large enough to maintain power in subsequent analyses and substantively meaningful in terms of identifiable qualitative group differences. The shape of the trajectories was another indicator of model selection. We examined models with nonlinear growth parameters (ie- quadratic, parabolic), dropping them from the model only in the case where these were nonsignificant.

Trajectory models were also estimated separately for each sex, and the resulting curve measurements were examined to identify potential differences in either the proportions of children in each group or their developmental patterns of BMI.

In order to verify how well individuals in our sample were classified into their respective trajectory groups, we examined descriptive data on the posterior probabilities of group membership [56, 72]. These probabilities are derived from model parameter estimates, range from 0–1, and give an indication of how much uncertainty was involved in classifying an individual into a particular trajectory given his or her data [73].

Bivariate analyses, including chi-square significance tests for categorical variables and ANOVA F-tests for continuous variables, were used to identify how early risk factors were distributed across trajectory groups. Factors for which the results of these analyses were significant at the p = 0.10 level were selected for inclusion in the multivariate model. Multinomial logistic regression analyses were then conducted in order to compare each of the early-onset vs. never overweight, late-onset vs. never overweight, and early-onset vs. late-onset overweight trajectory groups.

## Results

**Fig 1:**
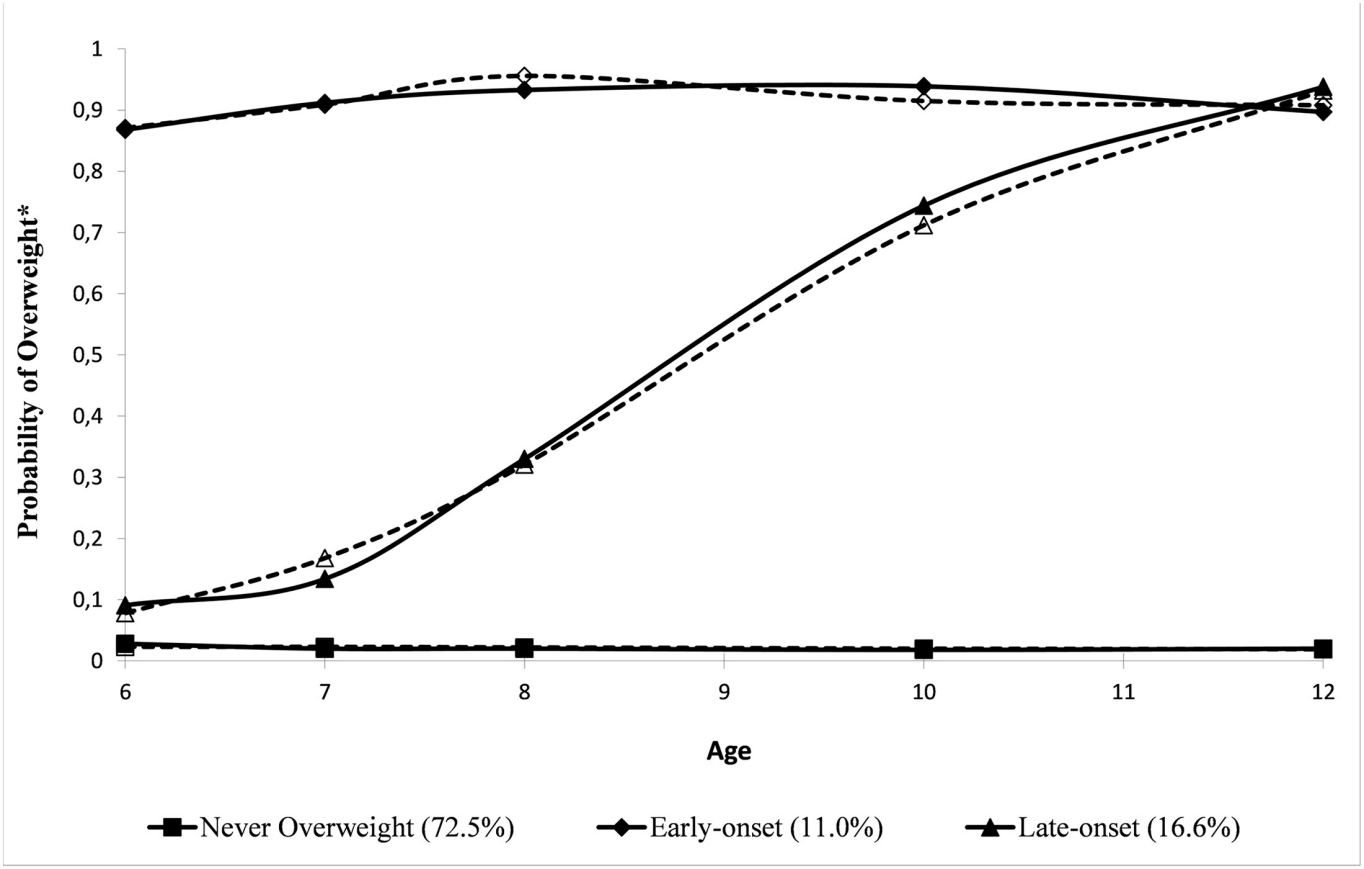
Group-based Trajectories of the Probability of Overweight from 6–12 yrs (n = 1678).

Fig 1 illustrates a model with three trajectories of weight status that was identified: “early-onset overweight” (11.0%), “late-onset overweight” (16.6%) and “never overweight” (72.5%). Solid lines represent observed values while dashed lines represent expected values. The early-onset group included children who had high probabilities of overweight throughout the assessment period, whereas the probability for overweight of the late-onset group began to rise at approximately age 7 to 8 years.

When modeled separately, the trajectory patterns were similar for boys and girls, although the proportions in each group differed. The fact that the patterns of development were similar indicated that a single model could be maintained, controlling for sex in subsequent analyses. The proportion of male children in each trajectory is listed in the first line of Table 2.

**Table 2:** Bivariate Associations Between Early Risk Factors and Overweight Trajectories from 6–12 years (n = 1552).

	Overweight Trajectories			p value^b^
Risk Factors [n, (%)] or [mean, (SD)^a^]	Never	Late-onset	Early-onset	
	n = 1103	n = 256	n = 192	
**Perinatal (gestation—1.5 yrs)**				
Male sex	520(47.1)	140(54.5)	83(43.2)	**0.04**
Size for gestational age				
average	903(83)	201(79.9)	150(78.7)	
small	100(9.2)	14(5.4)	13(6.9)	**0.0006**
large	84(7.7)	37(14.7)	27(14.4)	
Birth Rank				
1st	491(44.5)	102(39.8)	90(46.9)	
2nd	436(39.5)	118(46.1)	76(39.6)	0.33
3rd or more	176(16.0)	36(14.1)	26(13.5)	
Breastfed				
not at all	296(26.9)	65(25.6)	57(30.5)	
< 3 mo.	267(24.3)	68(26.8)	51(27.3)	0.49
> = 3 mo.	538(48.9)	121(47.6)	79(42.2)	
Average infancy weight gain (birth-1.5 yrs.), g^a^	468.99(81.81)	491.76(82.88)	510.89(92.32)	**<0.001**
Mother smoked during pregnancy	266(24.2)	76(29.6)	65(34.0)	**0.008**
Mother had gestational diabetes	242(21.9)	58(22.7)	50(25.9)	0.48
Parental Overweight				
no parent overweight	477(43.5)	64(25.8)	43(23.0)	
one parent overweight	508(46.3)	109(44.0)	83(44.4)	**<0.001**
both parents overweight	112(10.2)	75(30.2)	61(32.6)	
**Early Childhood (5 mo–5 yrs)**				
Preschool history of overweight (2.5–5 yrs)	123(11.2)	62(24.2)	125(65.1)	**<0.001**
Childcare (1.5–4yrs.)				
Parental Care	187(18.9)	50(21.6)	35(20.3)	
Daycare	275(27.8)	81(35.1)	55(32.0)	0.16
Relative	122(12.3)	27(11.7)	18(10.5)	
Non-relative	405(41.0)	73(31.6)	64(37.2)	
Aggressive behaviour (2.5–5 yrs)^a^	1.87(1.08)	2.08(1.24)	1.94(1.12)	**0.02**
DAS symptoms (2.5–5 yrs)^a^	1.24(0.92)	1.38(1.10)	1.19(0.85)	**0.05**
Mean Difficult Temperament (5 mo., 1.5 yrs)^a^	2.55(1.31)	2.62(1.36)	2.56(1.26)	0.75
Peer victimization, average score from 3.5–6 yrs^a^	1.60(1.19)	1.92(1.35)	1.69(1.32)	**0.001**
Slept < 10 hrs per night, average from 2.5–5 yrs	274(25.2)	61(24.0)	70(36.3)	**0.004**
Television viewing, average hours per week^a^	11.37(5.47)	12.38(5.57)	13.00(6.90)	**<0.001**
Sports lessons (with instructor) at age 5				
once per week or more	280(25.9)	59(23.0)	46(23.8))	
one or two sessions	104(9.4)	26(10.1)	15(7.8)	0.81
about once per month or less	719(65.2)	172(66.9)	132(68.4)	
Dance/gymnastics/martial arts lessons at age 5				
once per week or more	244(22.1)	54(21.0)	44(22.8)	
one or two sessions	94(8.5)	21(8.2)	11(5.7)	0.26
about once per month or less	765(69.4)	182(70.8)	138(71.5)	
Free-time physical activity at age 5	204(18.5)	42(16.4)	31(16.1)	0.59
(less than once per week vs. more)				
**Family and social environment (5 mo–5 yrs)**				
Young mother (<21)	161(15.1)	53(21.3)	46(24.3)	**0.002**
Mother's lifetime depression occurence	258(23.8)	49(19.4)	38(20.0)	0.20
Mother's antisocial history^a^	0.79(0.89)	0.90(1.02)	0.84(0.99)	0.25
Maternal affection towards child^a^	9.66(0.72)	9.68(0.62)	9.71(0.65)	0.61
Maternal perceived impact^a^	8.29(1.55)	8.22(1.66)	7.83(2.06)	**0.002**
Maternal efficacy^a^	8.51(0.97)	8.55(1.10)	8.53(1.03)	0.89
Maternal overprotection^a^	4.38(2.05)	4.68(2.14)	5.04(2.11)	**<0.001**
Maternal hostile/aggressive parenting ^a^	2.93(1.62)	3.01(1.58)	2.67(1.41)	**0.07**
Mother did not obtain high school diploma	192(17.4)	61(23.7)	51(26.6)	**0.002**
Family chronic poverty trajectory	148(13.4)	49(19.1)	42(21.9)	**0.001**
Transient poverty trajectory	162(14.7)	45(17.6)	37(19.3)	
Family ever food insufficient (1.5, 4.5 yrs)	60(5.5)	26(10.2)	18(9.6)	**0.006**
Family not intact, ever (from 5 mo.-5 yrs)	362(32.8)	101(39.3)	79(41.1)	**0.02**
Level of family functioning^a^	1.54(1.28)	1.66(1.28)	1.77(1.22)	**0.04**
Neighborhood Social cohesion^a^	1.83(0.45)	1.90(0.45)	1.89(0.45)	**0.06**
Neighborhood Social problems^a^	1.17(0.25)	1.24(0.30)	0.21(0.27)	**<0.001**
Immigrant status	127(11.5)	31(12.1)	41(21.4)	**0.001**

^a^ frequency (n) and percentage (%) provided for categorical variables, mean and standard deviation (SD) for continuous/ordinal variables.

^b^ P value determined using x2 test(categorical variables) or analysis of variance F test (continous/ordinal variables).

Our analysis of the posterior probabilities for group membership revealed that less than 5% of the sample has a posterior probability of 0.65 or less. Moreover, 80% of the sample has a posterior probability of 0.997 or greater, indicating a good model fit [56].

Of the thirty-five variables analyzed in bivariate analyses (Table 2), twenty-three were associated with the BMI trajectories (p = 0.10 level) and included in the multinomial model (see bolded p-values, Table 2).

The final multinomial logistic regression analysis revealed the following risk factors to be statistically significant in the individual comparisons (Table 3). Comparing the “Early-onset” to the “Never” overweight trajectory groups: large size for gestational age (OR = 1.79), preschool overweight (OR = 11.32), shorter night sleep (OR = 1.66), two overweight parents (OR = 7.29), one overweight parent (OR = 2.22), overprotective parenting (OR = 1.12), and immigrant status (OR = 2.01). Comparing the “late-onset” to the “never” trajectory group: large size for gestational age (OR = 1.80), greater average infancy weight gain (OR = 1.003), preschool overweight (OR = 1.83), two parents overweight (OR = 5.01), one parent overweight (OR = 1.51), and the family having experienced food insufficiency (OR = 1.81). Comparing the “early-onset” vs. the “late-onset” trajectory group: male sex (OR = 0.63), preschool overweight (OR = 6.20), and shorter night sleep (OR = 1.68).

**Table 3:** Multinomial Regression Analyses of the Association Between Early Childhood Risk Factors and Group-Based Trajectories of the Probability of Overweight in Middle Childhood (n = 1408).

	Early-onset overweight^a^			Late-onset overweight^a^			Early-onset overweight^b^		
Risk factors	OR	95% CI	p-value	OR	95% CI	p-value	OR	95% CI	p-value
**Perinatal (<1.5 years)**									
Small for gestational age	0.85	(0.40–1.84)	0.68	0.64	(0.34–1.22)	0.18	1.33	(0.54–3.30)	0.54
Large for gestational age	**1.79**	(1.00–3.19)	0.05	**1.80**	(1.13–2.87)	0.01	0.99	(0.54–1.83)	0.98
Greater average infancy weight gain	1.003	(1.00–1.01)	0.03	1.003	(1.00–1.01)	0.003	1.00	(0.99–1.00)	0.84
Mother smoked during pregnancy	1.36	(0.86–2.14)	0.19	1.13	(0.78–1.63)	0.52	1.20	(0.73–2.0)	0.47
**Child (1.5–5 years)**									
Male sex	0.80	(0.54–1.20)	0.29	1.27	(0.92–1.74)	0.14	**0.63**	(0.40–0.99)	0.05
Overweight throughout preschool	**11.32**	(7.45–17.20)	<0.001	**1.83**	(1.23–2.72)	0.003	**6.20**	(3.83–10.03)	<0.001
Aggressive symptoms	1.03	(0.84–1.26)	0.79	1.03	(0.88–1.21)	0.70	1.00	(0.80–1.25)	0.98
Depressive and anxious symptoms	0.95	(0.75–1.21)	0.67	1.09	(0.92–1.30)	0.32	0.87	(0.67–1.13)	0.30
Victimized by peers	1.04	(0.86–1.25)	0.70	1.11	(0.96–1.28)	0.17	0.94	(0.76–1.13)	0.55
Shorter night sleep (<10 hrs)	**1.66**	(1.07–2.57)	0.02	0.99	(0.69–1.42)	0.94	**1.68**	(1.03–2.75)	0.04
Longer TV viewing, hrs/wV	1.02	(0.98–1.05)	0.31	1.00	(0.97–1.03)	0.72	1.02	(0.98–1.06)	0.24
**Family/environmental (1.5–5years)**									
Two parents overweight	**7.29**	(4.08–13.02)	<0.001	**5.01**	(3.24–7.74)	<0.001	1.46	(0.78–2.72)	0.24
One parent overweight	**2.22**	(1.34–3.68)	0.002	**1.51**	(1.04–2.19)	0.03	1.47	(0.82–2.62)	0.20
Young mother	1.60	(0.92–2.79)	0.1	1.09	(0.68–1.74)	0.72	1.47	(0.78–2.74)	0.23
Overprotective parenting	**1.12**	(1.01–1.25)	0.03	1.09	(1.0–1.125)	0.05	1.04	(0.92–1.17)	0.55
Hostile/Aggressive parenting	0.88	(0.77–1.01)	0.06	0.98	(0.88–1.09)	0.72	0.90	(0.77–1.04)	0.15
Maternal perceived impact	0.97	(0.86–1.11)	0.68	1.04	(0.93–1.16)	0.51	0.94	(0.81–1.08)	0.38
Chronic early poverty	0.97	(0.50–1.89)	0.92	0.98	(0.56–1.71)	0.93	0.99	(0.47–2.09)	0.98
Transient early poverty	0.86	(0.48–1.54)	0.61	1.04	(0.65–1.67)	0.86	0.82	(0.43–1.58)	0.55
Maternal low education	1.00	(0.57–1.74)	0.99	1.23	(0.79–1.93)	0.36	0.80	(0.44–1.50)	0.50
Family food insufficiency	1.35	(0.63–2.92)	0.45	1.81	(1.00–3.28)	0.05	0.75	(0.33–1.67)	0.48
Family not intact	1.10	(0.70–1.74)	0.68	1.06	(0.74–1.52)	0.77	1.04	(0.63–1.74)	0.87
Decreased family functioning	0.99	(0.83–1.19)	0.93	1.01	(0.88–1.16)	0.87	0.98	(0.81–1.20)	0.85
Dangerous neighborhood	0.90	(0.54–1.45)	0.64	0.96	(0.65–1.41)	0.84	0.92	(0.54–1.60)	0.78
Neighborhood social problems	1.21	(0.52–2.83)	0.67	1.70	(0.92–3.15)	0.09	0.71	(0.28–1.78)	0.46
Immigrant status	**2.01**	(1.05–3.84)	0.03	1.09	(0.62–1.90)	0.76	1.84	(0.88–3.86)	0.10

^a^: Compared to children who were part of the "Never Overweight" trajectory.

^b^: Compared to children in the late-onset trajectory.

OR: Odds ratio; CI: Confidence interval.

N.B: All values have been rounded to two decimal points. Bolded ORs indicate significant effects at the p = 0.05 level.

## Discussion

The first aim of this study was to model the developmental trajectories of overweight during middle childhood. Our results add to the growing literature aimed at identifying heterogeneous subgroups of weight status in the population [74] and help fill the gap in knowledge as to the patterning that occurs during the middle childhood to early adolescent period. We identified two distinct developmental trajectories leading to overweight by early adolescence. Comparisons between our results and existing studies are somewhat limited by differences in age range, time period under study, and method used for classifying overweight. However, certain similarities and differences can be drawn. Three previous studies identified similar patterns of heterogeneity in the development of overweight during middle childhood [11, 12, 14]. Our results are similar to Li and colleagues [11] in that we identified an “early” and “late” onset overweight group. The late-onset groups increased at approximately the same time period (~8 yrs), but differed in the proportion of children represented by that group (5.2% of the Li sample and 17.4% of our sample). This may be due to differences between this US and our Quebecois sample. For example, the trajectories under study were modelled over different time periods (1984–2000 in the Li et al study vs. 2004–2010 in our study) and differing methods were used in order to classify overweight (≥ 95th BMI percentile according to CDC growth charts in the Li et al. study vs. International Obesity Task Force cut points [47] in our study). Our results are also similar to Balistreri et al [12] and Garden et al [14] who distinguished between “early” vs. “late” onset overweight, using a binary overweight and a continuous BMI outcome measure, respectively. However, our developmental trajectories differ from studies that used BMI z-scores to trace their trajectories [13, 16, 17]. While increasingly common in the overweight literature, certain scholars have identified that these transformed scores may be problematic for longitudinal use [48, 75] and for group-based trajectory models in particular [15]. For example, for two children measured at the same two ages, a one-unit BMI z-score change may represent very different changes in actual BMI [75]. Moreover, the measure performs differently depending on the baseline weight status of the child [48]. When used longitudinally in group-based developmental trajectory modeling, these may not allow the identification of distinct groups with various developmental patterns [16, 76], since trajectories seek to distinguish between typical and atypical patterns of development and BMI z-scores reduce the variability of extreme scores [48].

The second aim of the study was to identify early risk factors for the trajectories leading to overweight at the start of adolescence. To our knowledge, this is the first time a comprehensive set of putative early childhood risk factors was used to achieve this aim. We examined 3 types of risk factors: those related to the perinatal period; those specific to the child, and those belonging to the family and social environment. The bivariate analyses indicated that 23 factors were significantly associated with the developmental trajectories. Multivariate analyses reduced the number of significant risk factors to 8. Among the risk factors associated with the two overweight trajectories, approximately half were common to the early and late-onset groups and the other half were specific to either group. Specifically, we found that preschool overweight, parent overweight, and large size for gestational age were common risk factors. Preschool overweight was highly associated with the “early-onset overweight” trajectory (OR = 11.32), which corroborates the literature regarding the tracking of overweight from early to later childhood [35, 77, 78]. Compared to one overweight parent, two overweight parents approximately tripled the odds of the child being overweight at the beginning of adolescence. The importance of parent overweight is also corroborated by the literature, and may represent each of genetic, epigenetic, and family environment factors which have yet to be disentangled [32, 79–81]. Large size for gestational age was also identified as increasing the risk for membership in the early and late trajectories by nearly two-fold. This result coincides very closely with the aforementioned study by Li and colleagues [11], who identified high birth weight as a significant predictor of each of an early and late trajectory of overweight (also with an OR = 2). A baby’s birth weight is thought to be a marker for various perinatal and prenatal influences, including processes implicating a mother’s lifetime nutritional status or again by stress or other hormones secreted during gestation [9]. Controlling for these three important risk factors, we also identified risk factors that were specific predictors of the differing patterns of overweight development. Shorter nighttime sleep duration, immigrant status and to a lesser extent, overprotective parenting, were specific predictors of the early-onset trajectory. Numerous other studies point to sleep in early childhood as a risk factor for overweight by 6 yrs [36, 60, 82–85]. Possible underlying mechanisms include increased caloric intake due to hormonal mechanisms and increased opportunities for eating, as well as reduced energy expenditure resulting from altered thermoregulation and increased fatigue [84, 86].

Our results also concur with other research suggesting that children of immigrant mothers are at higher risk for overweight than children of non-immigrant mothers [87], including Balistreri et al.[12], who also identified immigrant status was associated with an early, as opposed to late, onset of overweight.

Previous research has found effects of parenting on children’s weight profiles [10, 41, 88]. For example, “controlling” mothering has been linked to an increased likelihood of child overweight between 4.5 and 12 years [10]. We found that mothers’ overprotective parenting in early childhood was significantly associated with a slightly increased odds a child would follow an early-onset overweight trajectory. This association has been hypothesized as being due to decreased opportunities for physical activity among children who experience overprotection [89].

Family food insufficiency at 1.5 and/or 4.5 years increased the likelihood that a child followed the late-onset, but not the early-onset trajectory by nearly two-fold, albeit at a borderline significance level. There is substantial evidence linking food insecurity or insufficiency with child overweight, although the results in the current literature are mixed [90, 91]. Moreover, it is difficult to disentangle this concept from related measures of poverty and the mechanisms linking it with child overweight may be varied. Families who do not have sufficient resources to buy food may have no choice but to rely on more energy-dense nutrition [92]. If this problem was present for the mother during pregnancy, the latent effect suggested by our study may support the thrifty phenotype hypothesis, whereby undernutrtion during perinatal and infancy periods “programs” changes in the metabolic system, predisposing a child to later overweight [93–95]. This is speculative however, and further studies examining food insecurity during pregnancy and trajectories of weight status would be needed to verify this.

### Strengths and Limitations

This study’s strengths include its large population-based sample, measured height and weight, and the ability to control for a wide variety of risk factors longitudinally. As maternal BMI and food insufficiency were only measured from 1.5 years after childbirth, some limitations include the fact that we cannot draw conclusions about the influence of maternal overweight or undernutrition during gestation. Nor can we distinguish the effects of heritability of adiposity status from common environmental effects. Finally, it is important to note that the group based developmental trajectories method is a useful statistical tool for modelling development in a general fashion, and for identifying the risk factors potentially associated. The risk factors we identified are not necessarily causes of overweight. Other unexamined variables may explain one or more of the observed associations.

## Conclusion

From a large set of risk factors we identified three that are common for both early and late onset overweight: parental overweight and overweight during the preschool years and large size at birth for gestational age. Three risk factors were specifically associated with the early onset of overweight: short sleep duration, maternal overprotection, and immigrant status. One was specifically associated to late-onset overweight: shorter gestational age and family food insufficiency in early life. These results suggest the early prevention of childhood overweight should specifically target families of pregnant women with the following characteristics: at least one parent overweight, the family is likely to suffer from food insufficiency, immigrant families. Two other risk factors suggest that postnatal preventive interventions targeting parenting skills related to overprotection and children’s sleep patterns may also be warranted.
